# Big stride in gene therapy for hemophilia B in China

**DOI:** 10.1097/BS9.0000000000000150

**Published:** 2023-01-13

**Authors:** Jianmin Wang, Dan Yang

**Affiliations:** a Department of Hematology, Shanghai Changhai Hospital, Shanghai 200433, China; b Shanghai Daopei Hospital for Hematological Disorders, LuDaoPei Medical Group, Shanghai 201112, China

In a recent issue of *Lancet Haematology*, Xue et al^1^ reported an adeno-associated virus (AAV)-based gene therapy in 10 patients with hemophilia B (HB) from China. BBM-H901, a novel vector comprised of an engineered liver-tropic AAV capsid (AAV843), synthesized liver-specific promoter and CpG reduced factor IX (FIX) Padua coding sequence, was infused in 10 patients (baseline FIX coagulation activity [FIX:C] were less than 2 IU/dL) after 1 week of prophylactic prednisone pretreatment (1 mg/kg per day). After a median follow-up of 58 weeks, mean FIX:C reached 36.9 ± 20·5 IU/dL. No FIX inhibitors or serious adverse events were observed. All patients developed high titer neutralizing antibodies against vector capsid. The concentrations of alanine aminotransferase and aspartate aminotransferase in plasma were below the upper limit of normal range in 8 patients. No FIX concentrate infusion was needed after gene therapy for these patients. This is a huge step forward in the treatment of HB in China.

Gene therapy with AAV vectors for hemophilia has been tried for dozens of years. Since 2003, gene transfer with a recombinant AAV (rAAV) vector expressing FIX was used as a method of treating HB through intramuscular injection. Eight adult men with severe HB (F.IX <1%) were enrolled in an open-label dose-escalation study. The circulating levels of FIX in all cases were less than 2% and most were less than 1% post-treatment, which were not sufficient to improve disease. This is the first report of an AAV vector gene therapy for HB. Although the level of FIX in vivo is unsatisfied, the results demonstrated that the AAV vector is safe in humans.^2^ To achieve long-lasting expressing of FIX in vivo, a modification of the protocol was reported in 2006 by the same researchers. The rAAV vector expressing human FIX was infused through the hepatic artery, instead of intramuscular injection. The new administration mode of vector could transduce human hepatocytes and resulted in therapeutically relevant levels of FIX, but a gradual decline in FIX was accompanied by a transient asymptomatic elevation of liver transaminases.^3^ Based on these data, a study focused on how to achieve stable therapeutic levels of FIX in vivo was published by Nathwani et al^4^ 5 years later. A single dose of an AAV8 vector expressing a codon-optimized FIX was infused through peripheral vein in 6 severe HB patients. This report has 3 major improvements which resulted in a 2% to 11% of normal levels of FIX in all patients: The codon-optimized AAV vectors can mediate FIX expression at higher levels; an AAV8 vector has a lower seroprevalence in humans than does AAV2; and AAV8 has a strong tropism for the liver, which is the reason the researchers infused the vector through the peripheral vein.^4^ For further verifying the safety and efficacy of the AAV8 vector, the long-term follow-up results of these 6 patients combined with 4 new HB patients’ data were reported by Nathwani et al^5^ in 2014. All 10 patients demonstrated an increase of FIX to a level that was 1% to 6% of the normal value over a median period of 3.2 years. No late toxic effect was reported.^5^ At that time, AAV-mediated gene therapy was known to result in therapeutic level but short-lived FIX circulation, or durable but suboptimal level of FIX.

To achieve durable high level of FIX, the vector dose should be increased. All studies above were based on using the high vector dose to arrive the effective level of FIX. How to use the relatively low dose of AAV vectors to achieving a stable expression of FIX while minimizing the risk of adverse events has become the main goal. In 2017, an AAV vector consisting of liver-specific promoter and FIX Padua (FIX–R338L) transgene was infused in 10 HB patients at a dose of 5 × 10^11^ vector genomes/kg (a relatively low dose). In this study, the sustained therapeutic expression of FIX was obtained in all patients with a mean FIX:C of 33.7 ± 18.5% (range, 14–81).^6^

A major challenge for safety and activity in gene therapy for HB was T cell immunity against AAV capsid proteins in the transduced hepatocytes or CpG oligodeoxynucleotides. Compared with other trials, there are 2 innovations in the article reported by Xue et al.^1^ One is the novel vector, BBM-H901. The cassette used in this study was optimized with CpG oligodeoxynucleotides ablation, thus avoiding immunity against CpG. Besides, a liver-specific promoter was combined with this CpG reduced FIX Padua coding sequence to enhance the liver tropism. AAV843, an engineered strong liver-tropic AAV capsid, can cause the rapid delivery of gene into liver. The vector DNA in plasma was found to be rapidly cleared by at least 3 logs within 24 hours. Rapid uncoating of the AAV843 capsid might favor rapid degradation of the capsid protein within the cells, and reduce the delayed T cell immune response against capsid protein-containing hepatocytes. The other important improvement is the prophylactic glucocorticoid treatment before vector infusion, as a pre-emptive preventive measure to reduce the potential immune response against AAV capsid and protect patients from delayed liver enzyme elevation induced by cellular immunity against transduced hepatocytes. Based on the success of this clinical study, a case report was just published by Xue et al in *N Engl J Med*.^7^ A 26-year-old man who had received gene therapy for HB underwent a successful total knee arthroplasty 16 months later. With a FIX level of 50 IU/dL, there was no excessive bleeding and no need for additional FIX infusion.

Looking back to the history, the first gene therapy for HB was published in China as early as 1990s. The human FIX gene was transferred ex vivo into skin fibroblasts by retroviral vector and injected subcutaneously in the abdomen of the patients repeatedly in months apart in 2 HB patients. The program was led by Professor Jing-Lun Xue in Fudan University in collaboration with our team led by Professor Peilin Meng in Changhai Hospital affiliated to the Second Military Medical University. The FIX:C rose from 2.19% to 5.92% and from 2.4% to 4.13%, respectively.^8^ After that, another 2 HB patients received the same retroviral vector gene therapy. This is also the first report of gene therapy for hemophilia in the world. The sustained expression of transgene at therapeutic level was not reached, but the plasma FIX levels were increased for more than 2-fold over 1 year.^9^ Although the protocols were complicated and laborious, and the expression level of FIX was low and not sustainable, it is indeed the milestone and pioneer of gene therapy for HB in China. Nearly 30 years have passed, the development of gene therapy for HB has made a great stride. AAV or lentiviral vector-mediated gene transfer or gene editing technologies are now entering clinical evaluation. In China, 3 clinical trials using AAV vectors for HB as investigational new drug approved by Center for Drug Evaluation have been initiated, including BBM-H901,^1^ VGB-R04, and ZS801, produced by 2 other different companies. In addition, a national registry for hemophilia was started in 2010 and a network for diagnosis and treatment of hemophilia was established in 2012, now comprises over 120 centers throughout the country. The progress is beginning to narrow the gaps in the management of patients with hemophilia between China and other countries.

The long-term expression of transgenic FIX at therapeutic levels is an important step toward development of curative gene therapy. Although the gene therapy for HB with AAV vector might be the mainstream in the future, several obstacles still remain, for example, immunologic response and liver toxicity, etc. Back to the article by Professor Lei Zhang and his colleagues, there are also lots of work to be done next. The long-term clinical benefit remains to be monitored for years and further evaluated in multiple center clinical trials. Additionally, the protocol of treatment deserves further exploration to establish the most optimized regimen, for example, the advantage of short-term glucocorticoid prophylaxis before vector infusion needs to be confirmed in a larger number of HB patients. The prospect is to eventually get the licensure of gene therapy for HB and benefit more patients with HB.
